# Exercise During Childhood Protects Against Cardiac Dysfunction Later in Life

**DOI:** 10.1093/geroni/igab046.2553

**Published:** 2021-12-17

**Authors:** Danielle Bruns, MacKenzie DeHoff, Aykhan Yusifov, Sydney Polson, Ross Cook, Emily Schmitt, Kathleen Woulfe

**Affiliations:** 1 University of Wyoming, Laramie, Wyoming, United States; 2 University of Colorado-Denver, Aurora, Colorado, United States

## Abstract

Cardiovascular disease continues to be a major cause of morbidity and mortality, particularly in aging populations. Exercise is amongst the most cardioprotective interventions identified to date, with early in life exercise such as during the juvenile period potentially imparting even more cardioprotective outcomes due to the plasticity of the developing heart. To test the hypothesis that juvenile exercise would impart later in life cardioprotection, we exercised juvenile male and female mice via voluntary wheel running from 3-5 weeks of age and then exposed them to cardiac stress by isoproterenol (ISO) at 4-6 and 18 months of age in adulthood and older age, respectively. We compared cardiac function and remodeling to sedentary control animals, sedentary animals who received ISO, and adult and aged mice that exercised for two weeks immediately before ISO exposure. Juvenile mice engaged in voluntarily wheel running, with male mice running 1.3 ± 0.8 km and female mice 2.8 ± 1.0 km a day. Echocardiography suggested that these juvenile animals underwent running-induced cardiac remodeling as evidenced by higher ejection fraction and stroke volume compared to sedentary controls. Exercise in the juvenile period attenuated ISO-induced cardiac hypertrophy and remodeling later in life compared to sedentary animals and those that exercised immediately before ISO administration. The mechanisms by which early versus late exercise is protective in adult and aged mice are under investigation. Further ongoing work will identify the adaptations induced by exercise in the juvenile heart that may help improve cardiac aging.

